# Nutrient Profile, Energy Digestibility in Pigs, and In Vitro Degradation Characteristics of Wheat Flour Milling Co-Products

**DOI:** 10.3390/ani15162460

**Published:** 2025-08-21

**Authors:** Rajesh Jha, Prajwal R. Regmi, Li F. Wang, Andrew Pharazyn, Ruurd T. Zijlstra

**Affiliations:** 1 Department of Human Nutrition, Food and Animal Sciences, University of Hawaii at Manoa, Honolulu, HI 96822, USA; 2 Department of Agricultural, Food and Nutritional Science, University of Alberta, Edmonton, AB T6G 2P5, Canada; 3 Center for Veterinary Medicine, United States Food and Drug Administration, Rockville, MD 20855, USA; 4 Trouw Nutrition Canada, Puslinch, ON N0B 2J0, Canada

**Keywords:** digestible energy, fermentation, in vitro, pig, wheat co-product

## Abstract

Using wheat flour milling (WFM) co-products in pig diets may reduce feed cost. Still, energy digestibility is lower for WFM co-products than for feed grains. Inadequate information exists about their fermentation characteristics and the relationship between digestible energy (DE) value and chemical characteristics or in vitro energy digestibility. The objectives were to (1) determine the chemical characteristics, in vitro and in vivo DE values, and energy digestibility of WFM co-products in growing pigs; (2) determine their in vitro microbial fermentation characteristics, and (3) establish relationships between in vivo DE value of WFM co-products and their chemical composition, fermentation characteristics, or in vitro digestibility values. Across Canada, 94 WFM co-products were sampled and characterized for their chemical composition and in vitro dry matter (DM) and energy digestibility using pepsin, pancreatin, and a multi-enzyme complex containing arabinase, β-glucanase, hemicellulase, xylanase, and cellulase. The in vivo energy, DM digestibility and DE value of 9 WFM co-products (2 shorts, 5 millrun, 1 middling, and 1 bran) were determined using a corn-based diet and 40 growing pigs in two periods to obtain 8 observations per diet. After in vitro digestion, the 9 WFM co-product samples were subjected to microbial fermentation using fresh fecal inoculum in a cumulative gas-production technique. The WFM co-products had a high content of crude fiber (up to 7.9% in shorts, 9.9% in millrun, 7.1% in middlings, and 12.0% in bran) and crude protein (CP; up to 27.8% in shorts, 20.0% in millrun, 22.1% in middlings, 15.9% in bran). The DE values ranged from 2.84 to 3.74 Mcal/kg DM among WFM co-products. Among chemical characteristics, neutral detergent fiber was the best predictor (R^2^ = 0.81) for in vivo DE value, followed by crude fiber (R^2^ = 0.78), and acid detergent fiber (R^2^ = 0.72). The in vitro DE values predicted (R^2^ = 0.80) in vivo DE values of 9 WFM co-products. Based on principal component analysis, total gas and short-chain fatty acid production varied among WFM co-products and was associated with the CP content of WFM co-products. In conclusion, WFM co-products contain high crude protein and have a high DE value for growing pigs but vary substantially in nutritional value.

## Simple Summary

During flour milling, a range of wheat flour milling (WFM) co-products are produced. Including these WFM co-products in pig diets may reduce feed cost; however, these co-products vary in their nutritional value. In total, 94 samples were analyzed for chemical characteristics. Of these, 9 samples were analyzed for digestible energy value in growing pigs, and for in vitro energy digestibility and fermentation characteristics. In conclusion, WFM co-products are interesting feedstuffs to consider for feed formulation. They contain high crude protein and have a high DE value for growing pigs, but vary substantially in nutritional value. The present study demonstrated that in vitro techniques are effective feed evaluation tools to accurately determine energy digestibility and fermentability of WFM co-products.

## 1. Introduction

Swine diet formulations are primarily based on cereal grains as the main energy source. With changes in feed market dynamics due to the availability and price of conventional feedstuffs, the feed industry is exploring the formulation of swine diets using alternative feedstuffs, including co-products from cereal grains [1,2]. However, alternative feedstuffs are typically rich in fiber and vary widely in their nutrient profile [3,4]. Thus, feed quality information is essential before including alternative feedstuffs into swine feeding programs.

During the production of wheat flour, the wheat milling industry generates various wheat flour milling (WFM) co-products that are generally classified according to their crude fiber content. For example, middlings contains less than 4% crude fiber, shorts 5 to 7%; millrun 8 to 10%, and bran contains more than 10% crude fiber. These WFM co-products are used in swine diets to ameliorate high feed cost, accounting for 65 to 70% of the total cost of pig production [5]. The WFM co-products are primarily composed of wheat seed coat, aleurone layer, germ, and small amounts of starchy endosperm. Thus, WFM co-products are enriched in crude protein (CP), fiber, minerals, and fat and contain less starch than the parent wheat grain [6]. Fiber and protein influence digestibility and fermentability of plant-based co-products in pigs [7,8] that may consequently affect growth performance and health of pigs [9]. Information about the nutrient profile and digestion and fermentation characteristics may support the use of co-products in pig diets and modulation of gut environment and nutrient management [8]. The digestible energy (DE) value among WFM co-products in pigs can vary widely and ranged from 2.61 to 2.99 Mcal DE/kg of dry matter (DM) among 5 samples [10]. Therefore, before incorporating WFM co-products into a swine diet, the prediction of the DE value of the WFM co-product is important to ensure that the targeted dietary DE value is achieved. Methods to predict the DE value of other feed ingredients, such as barley and wheat, in pigs have been previously reported [11,12,13], but a method to predict the DE value of WFM co-products is lacking.

We hypothesized that in vitro digestion techniques can accurately predict energy digestibility of WFM co-products [14]. The objectives of the present study were to: (1) determine chemical characteristics, in vitro and in vivo DE value, and digestibility of WFM co-products using a corn-based diet in growing pigs, (2) establish the relationship between chemical composition and energy digestibility of WFM co-products, and (3) characterize WFM co-product samples microscopically and determine their in vitro fermentation characteristics.

## 2. Materials and Methods

The WFM co-product samples (*n* = 94) were collected across Canada by Trouw Nutrition Canada Inc. (Puslinch, ON, Canada) and were characterized for their chemical composition. The animal protocol was approved by the University of Alberta Animal Care and Use Committee for Livestock, and followed principles established by the Canadian Council of Animal Care [15]. The animal experiment was conducted at the Swine Research and Technology Centre (SRTC) of the University of Alberta, Edmonton, AB, Canada.

### 2.1. Experiment 1. In Vitro Digestibility

The in vitro DM and energy digestibility of 94 WFM co-product samples were determined using an established technique [11]. Briefly, a sample (0.5 ± 0.1 g) was weighed into a 125 mL conical flask. Phosphate buffer (25 mL, 0.1 N, and pH 6) solution was added to the flask and stirred using a small magnetic rod. After adding 10 mL of 0.2 N HCl solution to the flask, the pH of the solution was adjusted to 2 using 1 N HCl or 1 N NaOH solutions. Then, 1 mL (25 mg/mL) of freshly prepared pepsin (P-7000, Sigma-Aldrich, St. Louis, MO, USA) and 0.5 mL chloramphenicol solutions (0.5 g/100 mL of ethanol) were added to the flask. The flask was incubated in a water bath at 39 °C for 2 h. After the incubation, 10 mL of 0.2 N phosphate buffer (pH 6.8) and 5 mL of 0.6 N NaOH solutions were added to the flask, and the pH of the solution was adjusted to 6.8 with 1 N HCl or 1 N NaOH solution. Thereafter, 3 mL (100 mg/3 mL) of freshly prepared pancreatin (P-1750; Sigma-Aldrich) solution was added to the flask. The flask was incubated in a water bath at 39 °C for 4 h. After the second incubation, 10 mL of a 0.2 M EDTA solution was added to the flask, and the pH was adjusted to 6.8 with 30% acetic acid solution. Then, 0.5 mL of Viscozyme (multi-enzyme complex from *Aspergillus aculeatus* containing arabinase, β-glucanase, hemicellulase, xylanase, and cellulase; Novozymes, Bagsvaerd, Denmark) was added, and the flask was incubated at 39 °C for 18 h. The Viscozyme supplied ≥ 100 β-glucanase units/g of product [12,13].

The enzymatic digestion was terminated by the addition of 5 mL of 20% sulphosalicylic acid, and the flask was kept at room temperature for 30 min to facilitate precipitation of undigested soluble proteins. The undigested residue was then collected in a filtration unit using a porcelain filtration funnel lined with pre-weighed filter paper (Whatman no. 54; Whatman Inc., Florham Park, NJ, USA). The residue, along with the filter paper, was dried overnight at 80 °C [12,13].

### 2.2. Experiment 2. In Vivo Digestibility

Ten experimental diets were used in the animal study; 9 corn-based diets included WFM co-product samples (2 shorts: Shorts A and Shorts B; 5 millrun: Millrun A to Millrun E; 1 middlings; and 1 bran) and a diet containing solely corn as energy source (Table 1). The corn was ground using a hammer mill (Jacobson, Carter Day International, Minneapolis, MN, USA) with an 8/64”-screen for diet mixing. The WFM co-product samples were not ground as the particle size was smaller than the ground corn. The average values of ingredient composition from NRC (2012) [16] were used in diet formulation. The individual WFM co-product sample and corn were presumed to be the only sources of energy in the diets, and the small contribution of energy from the vitamin and mineral premixes was assumed to be negligible. Diets were fed as mash and were fortified to meet or exceed the vitamin and mineral requirements of 20- to 50 kg growing pigs [16]. Chromic oxide was used as an indigestible marker [10].

In total, 40 pigs [Camborough-22 × Line 65, PIC Canada Ltd., Airdrie, AB, Canada; 37.5 ± 2.2 kg of initial body weight (BW)] were each used in 2 consecutive periods in 2 replications of 20 pigs, for a total of 80 pig observations or 8 pig observation per diet. Each pig within a replication was assigned randomly to 1 of the 10 test diets during period 1 and a different test diet during period 2. Each of the pigs fed the same diet during period 1 received a different diet from each other during period 2. Pigs were housed in individual pens that allowed freedom of movement for 30 d starting with a 10 d acclimation to a standard SRTC pre-grower diet, followed by 2 consecutive 10 d experimental periods. Each experimental period comprised a 5 d adaptation to a specific experimental diet, followed by a 5 d collection of feces [12,13]. Daily feed allowance was adjusted to 3 times the maintenance requirement for energy (3 × 110 kcal of DE/kg of BW^0.75^) [17]. Pigs received an equal amount of feed twice daily at 0800 and 1530 h. Pigs had free access to water throughout the experiment [12,13].

Freshly voided feces were collected hourly from 0800 to 1530 h by using the grab method. Feces were pooled for each pig and frozen at −20 °C. Before analyses, feces were thawed, homogenized, subsampled, and freeze-dried [12,13].

### 2.3. Experiment 3. In Vitro Fermentation

Six WFM co-product samples (2 shorts: A and B; 2 millrun: B and D; 1 middlings and 1 bran) were selected from the samples used in Experiment 1 (Section 2.1), based on the diversity in their fiber and crude protein content (Table 2) and their ATTD of GE in pigs (Table 3). The WFM co-product samples were subjected to microbial fermentation with the following experimental design: [(6 WFM co-product × 6 replicates) + 6 blanks} × 2 batches], yielding 12 observations per WFM co-product.

The WFM co-product samples underwent in vitro pepsin and pancreatin hydrolysis, following the first 2 steps of the protocol of Boisen and Fernandez (1997) [11], as described in Experiment 2 above (Section 2.2). After the second step, the residues were collected by filtration using a nylon cloth (42 µm), washed with ethanol (2 × 25 mL 95% ethanol), and acetone (2 × 25 mL 99.5% acetone), dried for 24 h at 60 °C and weighed. For each WFM co-product sample, the enzymatic hydrolysis was repeated 14 to 16 times, depending on each sample’s degradability to obtain enough undigested residue for the in vitro fermentation. The CV of in vitro DM disappearance (IVDMD) during hydrolysis within and between the batches for each ingredient ranged from 0.09 to 2.84%. Hydrolyzed residues from the different replicates and batches of the same WFM co-product were pooled for subsequent use in the in vitro fermentation.

The rate of fermentation of the substrates was assessed in vitro, using a cumulative gas-production technique adapted to the pig [18,19]. Briefly, 200 mg samples were incubated at 39 °C in a shaking water bath at 50 rpm in a 125 mL-glass bottle with 30 mL buffer solution containing macro- and micro-minerals [20], and a fresh fecal inoculum. Three growing pigs (6–8 weeks age) from the herd of the SRTC (University of Alberta, Edmonton, AB, Canada), fed a standard commercial diet devoid of antibiotics, were used as donors for the fecal inoculums. Fecal samples were collected directly from the rectum and immediately placed in airtight plastic syringes and kept in a water bath at 39 °C until further use within 1 h. The inoculum prepared from feces was diluted 20 times in the buffer solution with NaHCO_3_ and (NH_4_)HCO_3_ and filtered through a 250 µm-screen and then transferred into the bottle with fermentation substrates. Bottles were sealed with a rubber stopper and placed for incubation. An anaerobic environment was maintained throughout the experiment, from the inoculum preparation until the incubation step by flushing with CO_2_ gas. The gas generated by the fermentation process and the CO_2_ released by the buffering of the short-chain fatty acids (SCFA) produced during the fermentation were measured at 0, 2, 5, 8, 12, 18, 24, 36, 48, and 72 h by means of a pressure transducer (GP:50 SIN-54978, Grand Island, NY, USA) [21], fitted with digital data tracker (Tracker 211, Intertechnology Inc., Don Mills, ON, Canada). The bottles were vented after every measurement. The fermentation was stopped at 72 h of incubation by quenching the bottles in iced water.

At the end of the fermentation, samples were collected from the bottles for measurement of SCFA [18,19]. Samples of inoculum prior to fermentation were analyzed for SCFA. Gas measured at a different time point during fermentation was modeled to obtain the fermentation kinetics and total gas production. The fermented solution was centrifuged. The liquid phase of the fermented solution was taken out quantitatively and subjected to SCFA analysis using GC.

### 2.4. Chemical Analyses and Calculations

The WFM co-products, test diets, and freeze-dried fecal samples were ground in a Retsch mill (model ZM1, Brinkman Instruments, Rexdale, ON, Canada) through a 1 mm screen and analyzed for DM by drying at 135 °C in an airflow-type oven for 2 h (method 930.15) [22]. The gross energy (GE) of WFM co-products, diets, and fecal samples was analyzed by an adiabatic bomb calorimeter (Model 5003, IKAWerke GmbH and Co. KG, Staufen, Germany); benzoic acid was used as a standard. Chromic oxide in feed and feces was analyzed by a spectrophotometer (LKB-Ultraspec III model 80-2097-62, Pharmacia, Cambridge, UK) at 440 nm after ashing overnight at 450 °C [23]. Furthermore, the WFM co-product samples were analyzed for the content of CP (Kjeldahl N; method 990.03) [22], acid detergent fiber (ADF), neutral detergent fiber (NDF) [24], ether extract (method 920.39) [22], and ash (method 942.05) [22].

The concentrations of SCFA in the post-fermentation solution were determined using GC with a method used by Jha et al. (2011) [18,19]. Briefly, 0.8 mL of the test sample (supernatant from centrifuged at 2500× *g* for 10 min at 4°C) was added in a tube with 0.2 mL of 25% phosphoric acid and 0.2 mL of internal standard solution (150 mg of 4-methyl-valeric acid, S381810, Sigma-Aldrich) and vortexed thoroughly. The sample was analyzed for SCFA (i.e., acetate, propionate, butyrate, iso-butyric, valeric, iso-valeric, and caproic acids) using a Varian model 3400 gas chromatograph (Varian, Walnut Creek, CA, USA) with a Stabilwax-DA column (30 m × 0.25 mm i.d.; Restek, Bellefonte, PA, USA). A flame-ionization detector was used with an injector temperature of 170 °C and a detector temperature of 190 °C. Branched-chain fatty acid (BCFA) content was calculated as the sum of iso-butyric acid and iso-valeric acid.

### 2.5. Scanning Electron Microscopy

The 6 WFM co-product samples used in the Experiment 3 (Section 2.3) were attached on circular aluminum stubs with double-sided tape, coated with gold to a thickness of 12 nm, and photographs were taken at an accelerating voltage of 5 kV with an FXV scanning electron microscope (model JSM 6301, JEOL, Tokyo, Japan).

### 2.6. Kinetics of Gas Production

Gas pressure measurements were converted into gas volume (*G*, g^−1^DM) using the ideal gas law, assuming an atmospheric pressure of 101,325 Pa and a temperature of 312.15 K. Gas accumulation curves recorded during the 72 h of fermentation were modeled according to France et al. (1993) [25]:

(1) $$\begin{array}{c} G\left(mLg^{-1}DM\right)=0,\;if\;0<t<L\\ =G_{f}\left(1-exp\left\{-\left\langle b\left(t-L\right)\right.+\left.c(\sqrt{t}-\sqrt{L})\right\rangle \right\}\right),\;if\;t\geq L \end{array}$$

where *G* denotes the gas accumulation to time, *G_f_* (mL g^−1^DM) the maximum gas volume for *t = ∞* and *L* (h) the lag time before the fermentation starts. The constants *b* (h^−1^) and *c* (h^−1/2^) determine the fractional rate of degradation of the substrate *µ* (h^−1^), which is postulated to vary with time as follows:

(2) $$\mu =b+\frac{c}{2\sqrt{t}},\;if\;t\geq L$$

Kinetics parameters (*G_f_*, *L*, *µ_t_ = _T/_*_2_ and *T*/2) were compared in the statistical analysis. *T*/2 is the time to half-asymptote when $G=G_{f}/2$.

### 2.7. Calculation of Digestibility

Based on the results of the chemical analyses, the apparent total tract digestibility (ATTD) of energy for each diet was determined using the indicator method [26]. The ATTD of energy of the specific WFM co-product sample was calculated by difference from the corn diet [27]. The DE value was calculated by multiplying the ATTD of energy by the GE content of the specific WFM co-product sample.

The in vitro energy digestibility was calculated using the following formula:

In vitro energy digestibility = [(sample DM × sample GE) − (residue DM × residue GE)]/(sample DM × sample GE) [18,19].

### 2.8. Statistical Analyses

To test the hypotheses, *p* < 0.05 was considered significant. In vitro digestibility of DM and energy of ingredients were subjected to ANOVA as randomized complete block design with sample replicate as experimental unit and batch as block using the MIXED procedure in version 9.4 of SAS [28]. The model included treatment (feedstuff) as the fixed effect and batch as the random factor.

For the animal study, the individual pig was considered as experimental unit. Digestibility of DM and GE of diets and energy digestibility of ingredients were compared using the MIXED procedure of SAS [28] with diet as the fixed effect and period as a random factor.

Fermentation characteristics of WFM co-product samples were compared using the MIXED procedure of SAS with the WFM co-product as the main factor and batch as a random factor. Means were separated using the Tukey method with a significance level of 0.05 and letter groupings was obtained using the SAS “pdmix800” macro [29].

Using the REG procedure of SAS [28], the R^2^ between the ATTD of energy and chemical characteristics was determined. The REG procedure was used to develop regression equations to predict the ATTD of energy based on in vitro DM and energy digestibility, using the R^2^ value to indicate the quality of the prediction equation [12,13]. The COR procedure of SAS [28] was used to determine correlations among variables. The WFM co-product sample was considered as the experimental unit for linear regression analyses

Principal component (PC) analysis was performed using JMP software in version 9.4 of SAS [28]. The physical and chemical characteristics of WFM co-product samples, energy digestibility from Experiment 2 (Section 2.2, and fermentation kinetics and SCFA profile from Experiment 3 (Section 2.3) were used as variables for PC analysis. The loading plots of PC 1 and PC 2, the first 2 eigenvalues, were used to determine correlations among the WFM co-product characteristics, fermentation kinetics, and SCFA profile. The angles between the lines were used to describe the interrelationship. In PC analysis, the length, direction, and angle between arrows indicates the correlation between variables or between variables and PC axes (e.g., α = 0° and r =1; α = 90° and r = 0; and α = 180° and r = 1). Percentages on X and Y axes indicate proportions of the variability of data that are described with the corresponding PC in the model.

## 3. Results

### 3.1. Chemical Characteristics

The WFM co-products varied widely in fiber and protein content within and among sample types (Table 2). Shorts A was more consistent with middlings, having a lower crude fiber and ADF content. Crude fiber for WFM co-products ranged from 5.2% in Shorts B to 12.0% in Bran, and CP ranged from 15.9% in Bran to 27.8% in Shorts A. Both ADF and NDF content were lowest in Shorts B (8.0 and 22.9%, respectively) and greatest in Bran (15.5 and 49.2%, respectively). The GE value was lowest for Bran and greatest for Middlings.

### 3.2. Energy Digestibility

The in vivo DE value of diets and ATTD of GE differed among (*p* < 0.001; Table 3) and within WFM co-product. The Diet DE value was greatest for Short B and lowest for Bran (3.56 and 3.21 Mcal/kg DM, respectively). The DE value of Millrun varied from 3.30 to 3.40 Mcal/kg DM. The ATTD of GE ranged from 74.4% in Bran to 82.5% in Shorts B, and the ATTD of GE of corn diet was 82.9%.

The in vitro DM and GE digestibility differed (*p* < 0.001; Table 3) among wheat co-products. Shorts B (71.8% DM and 69.1% GE digestibility) and Middlings (71.7% DM and 68.0% GE digestibility) had greater (*p* < 0.05) in vitro DM and GE digestibility than the other WFM co-products. Bran had the lowest (*p* < 0.05) DM and GE digestibility among the WFM co-products.

### 3.3. Relationship of Digestibility with Chemical and Fermentation Characteristics

**Figure 1:**
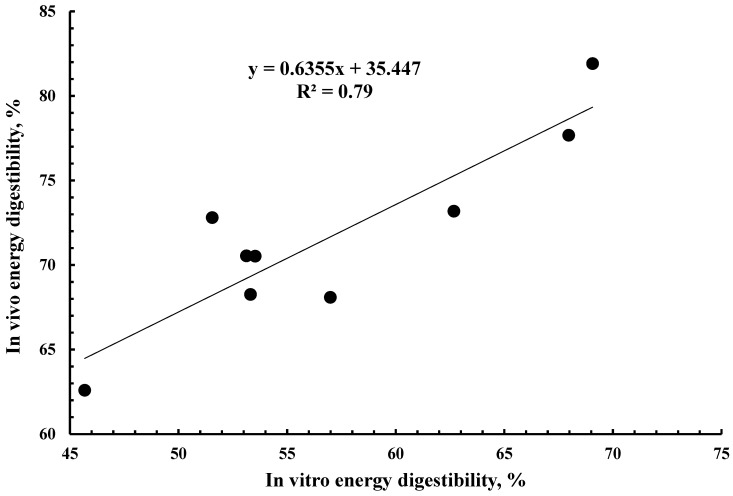
Relationship between apparent total tract digestibility of energy and in vitro energy digestibility of 9 co-products from wheat flour milling.

**Figure 2:**
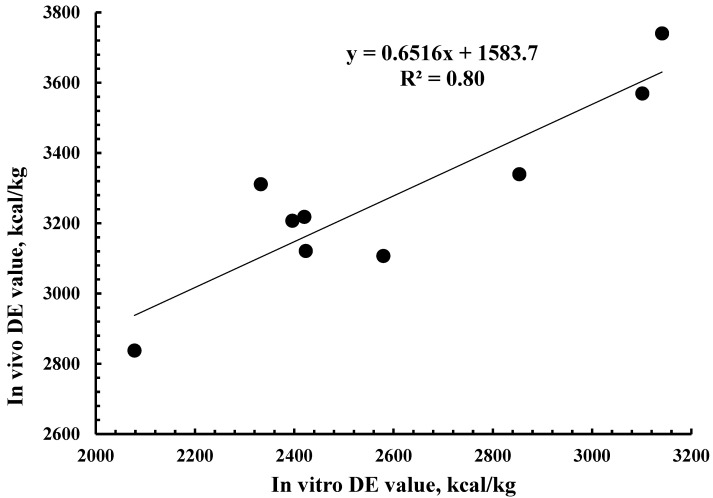
Relationship between in vivo digestible energy (DE) and in vitro DE value of the 9 co-products from wheat flour milling.

**Figure 3:**
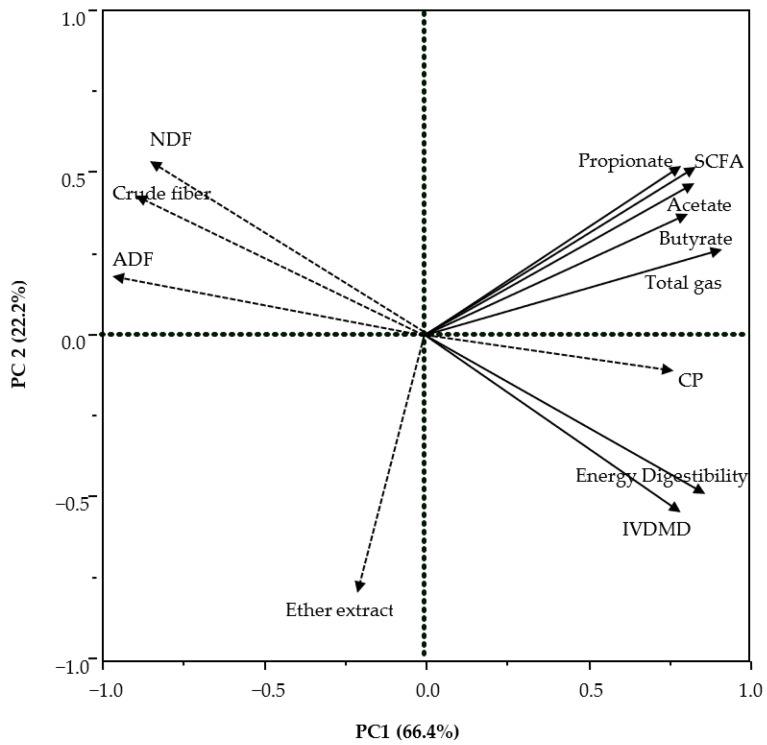
Loading plot from principle component (PC) analysis of co-products from wheat flour milling (WFM); Components showing the correlations among WFM co-product characteristics (dashed arrows), fermentation kinetics and SCFA profile studied in vitro (solid arrows) and in vivo energy digestibility and in vitro DM digestibility results (solid lines) of the first 2 eigenvalues (PC 1 and PC 2). IVDMD, in vitro dry matter digestibility measured after 2-stage enzymatic digestion. Energy digestibility after 3-stages in vitro enzymatic digestion.

The R^2^ between in vivo and in vitro energy digestibility was 0.79 (Figure 1). The R^2^ between in vivo and in vitro DE value was 0.80 (Figure 2). The R^2^ between in vivo DE value and chemical characteristics was greatest for NDF (0.81), followed by crude fiber (0.78), ADF (0.72), and CP (0.48) (Table 4). The principal component analysis (PCA) indicated that CP had a positive association with fermentation products, energy digestibility, and in vitro DM digestibility. Crude fiber, NDF, and ADF had a strong negative association with energy digestibility and in vitro DM digestibility (Figure 3).

### 3.4. In Vitro Fermentation Kinetics and Metabolites

The fermentation kinetics differed (*p* < 0.001; Table 5) among WFM co-products. The fractional rate of degradation was greatest (*p* < 0.05) for Bran and lowest for Shorts A. Bran had the longest (*p* < 0.05) lag time, and Shorts A took the longest (*p* < 0.05) time to reach half time of asymptote gas production. Total gas production was greatest (*p* < 0.05) for Middlings and Shorts A and lowest (*p* < 0.05) for Millrun D and was correlated negatively (r = −0.70 and −0.59, respectively; *p* < 0.001) with ADF and crude fiber. Total SCFA production had trends similar to total gas production. Total SCFA production ranged 1.0 mMol/g DM incubated among WFM co-products.

### 3.5. Microscopic Matrix Structure

**Figure 4:**
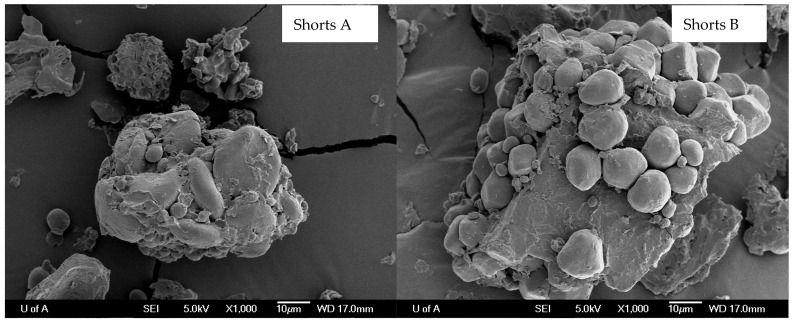

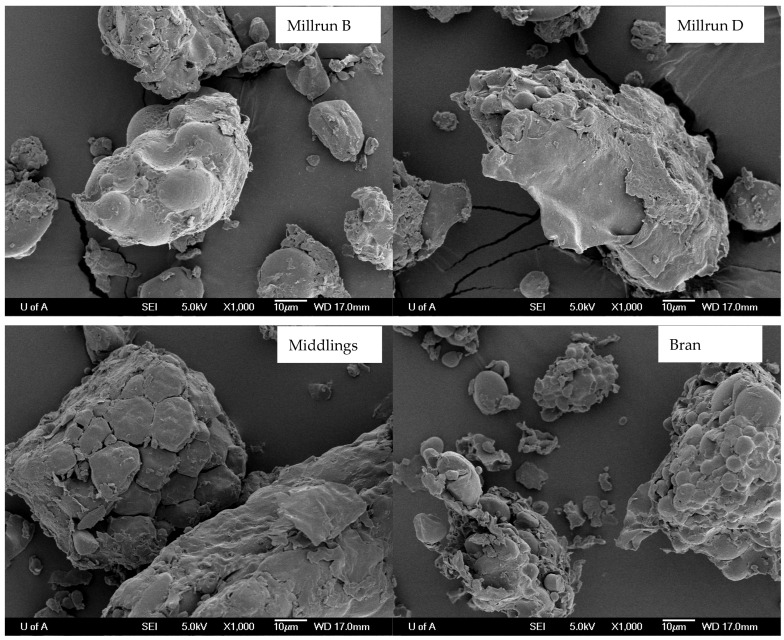
Scanning electron microscope images (×1000) of co-products from wheat flour milling.

The scanning electron microscopic images (Figure 4) revealed that both Shorts A and B contained more free starch granules and moderately loose starch-fiber matrix structure than Middlings and Brans. Instead, the starch granules in Millruns and Bran were trapped tightly within the fiber matrix.

## 4. Discussion

Alternative feedstuffs play a key role in ameliorating rapidly increasing feed costs [2]. The WFM co-products are widely available around the world and can be used in swine diets to develop cost-effective and sustainable feeding programs. The WFM co-products originate from the wheat milling industry that produces wheat flour for the human food industry [6]. Wheat consists of endosperm that is rich in starch, the germ that is rich in protein, fat, minerals, and vitamins, the aleurone layer that is rich in fiber and protein, and the seed coat that is rich in fiber [30,31]. During flour milling, the starch-rich endosperm is separated from the other parts of wheat grain. The isolated starch endosperm is then processed into flour, whereas the remaining parts of the wheat grain are processed into various co-products that are used mainly in the livestock feed industry. Thus, the WFM co-products contain more fiber and protein than the parent wheat grain [6]. The lack of information about nutritional compositions and digestibility value of WFM co-products limits their use in swine diets. For example, the analyzed crude fiber content of the WFM co-products used in the present study revealed that Shorts A and Middlings may contain more fiber than trade guidelines [32]. The present study indicated that the WFM co-products containing high CP and have greater DE values indicating that these can be used as an alternative feedstuff for supplying energy and perhaps even protein in swine diets to reduce feed cost, a major challenge of the swine industry [5]. Alternatively, the difference may reflect difference in the use by industry of the terms middlings, shorts and millrun, and that it is important to understand the fiber content of the WFM coproducts actually received compared to supplier definition.

The DE value among the WFM co-products ranged from 2.84 to 3.74 Mcal/kg. Therefore, before mixing a batch of WFM co-product into a swine diet, predicting its DE value is important to ensure that the targeted DE value of the diet is achieved. Previously, among chemical characteristics, ADF was the best predictor (R^2^ = 0.79) of in vivo energy digestibility for wheat samples with a wide range of physical and chemical characteristics [13]. In the present study, among chemical characteristics, NDF (R^2^ = 0.81) was the best predictor for in vivo DE value of WFM co-product samples, followed by crude fiber (R^2^ = 0.78) and ADF (R^2^ = 0.72). Greater fiber content was associated with reduced energy digestibly, as reflected by the lowest digestibility of Bran that contained most ADF, NDF, and crude fiber. This association between fiber content and energy digestibility is similar to previous findings when studying various feedstuffs [33,34,35,36]. The R^2^ value between in vivo DE value and in vitro DE value was greater in the present study for WFM co-product samples (0.80) compared to the R^2^ = 0.65 that was reported for wheat samples [13]. One reason for a greater R^2^ in the present study could be the greater range in energy digestibility of WFM co-product samples compared with wheat samples (11.2 vs. 19.3–units range) [13].

Differences in fermentation characteristics among WFM co-products can be attributed to their fiber and protein fractions, which are the key components influencing digestibility and fermentability of co-products in the pig intestine [8]. The range of total gas production from fermenting WFM co-products was similar to the range measured for wheat bran [18] and less than half of the total gas produced by wheat flour [19], with similar trends for the total SCFA production. This fermentation capacity seems to be associated with the carbohydrate-protein matrix and the type and amount of fiber contained in the WFM co-product samples [37,38]. The starch and proteins in the WFM co-products might be embedded within fiber (with some variation among WFM co-products), while the nutrient matrix of wheat is loosely embedded. This matrix complexity makes nutrients in WFM co-products less accessible to enzymes and microbes for digestion and fermentation [39]. The differences in the total gas and the SCFA produced among WFM co-product samples indicate that their fermentability varies. The difference is associated with their fiber and protein content, as indicated by a negative correlation between ADF and CP with DM degradability and total gas production. Indeed, greater fiber content was associated with delayed gas production and a greater proportion of acetate within the SCFA produced. Also, a study in pigs with such co-product feedstuffs, including wheat bran, revealed a similar correlation between nutrients and fiber degradation [8]. Variation existed in the amount of individual SCFA produced among WFM co-products, with the greatest amount of butyrate production in Shorts B, which contained the least ADF and NDF. This fermentation characteristic can be used to programming the gut health of pigs, including intestinal microbiota and immune functions [40]. Notably, the present in vitro model also has some limitations like any other. The digesta available for in vitro fermentation is not identical as in the large intestine of the pigs. Moreover, variation in the type and processing of feedstuffs may affect fermentation characteristics [18,19].

The nutrient composition of co-products and their digestibility and fermentability varied not only among WFM co-products but also within the same WMF co-product. However, instead of focusing on the specific co-product and its classification, it is essential to focus on the fiber content of the specific batch of WFM co-product, because fiber content will determine the digestibility and fermentability of the specific batch of WFM co-product. We also explored the nutrient matrix in detail using scanning electron microscopy. While microscopy reveals macrostructure of feed particles and does not specifically analyze macronutrients, the microscopic images revealed that the exposure of starch and protein granules from the fiber in the matrix differed among WFM co-product samples, showing direct associations with digestibility and fermentability. Loosely embedded nutrient matrices favor enhanced nutrient digestion and fiber fermentation [37]. Starch and protein trapped in the fiber matrix of the WFM may be released by using supplemental enzymes [37], thereby enhancing the degradation and fermentation of such WFM co-product samples in the pig intestine. Exogenous enzyme supplementation may maximize opportunities to include WFM co-products in swine diets and ameliorate reductions in nutrient digestibility [10].

## 5. Conclusions

In conclusion, WFM co-products vary in their nutrient profile, including their fiber content and in vitro fermentation profile within and among sample type. In addition, the WFM co-products vary in their in vivo DM and energy digestibility in swine. Chemical characteristics such as ADF content and in vitro digestibility value can be used to predict the in vivo DE value of WFM co-products. Also, in vitro degradation and fermentation characteristics vary among WFM co-products and this variation is mainly associated with the fiber and protein content. Therefore, treatments targeted to reduce the impact of fiber and to increase protein content may increase the digestibility and fermentability of WFM co-products. Thus, the type and composition of WFM co-products should be considered for pig diet formulation. The WFM co-products can serve as an alternative feed ingredient as these co-products from wheat flour milling contain high crude protein and DE value for growing pigs. Finally, the present study also validated that in vitro techniques are effective feed evaluation tools that can be used to predict energy digestibility and fermentability of similar cereal-based feedstuffs accurately.

## Figures and Tables

**Table 1:** Ingredient composition (g/kg) of the experimental diets for in vivo determination of apparent total tract energy digestibility of co-products from wheat flour milling.

Ingredient, %, As-Fed Basis	Test Diet	Control Diet
Corn	560	960
WFM co-products ^1^	400	–
Limestone	12	12
CaHPO_4_	8	8
Salt	5	5
Mineral premix ^2^	5	5
Vitamin premix ^3^	5	5
Chromic oxide	5	5

^1^ WFM, wheat flour milling. ^2^ Provided per kilogram of diet: vitamin A, 8250 IU; vitamin D_3_, 825 IU; vitamin E, 40 IU; niacin, 35 mg; d-pantothenic acid, 15 mg; riboflavin, 5 mg; menadione, 4 mg; folic acid, 2 mg; thiamine, 1 mg; D-biotin, 0.2 mg; and vitamin B_12_, 0.025 mg. ^3^ Provided per kilogram of diet: zinc, 100 mg as zinc sulfate; iron, 80 mg as ferrous sulfate; copper, 50 mg as copper sulfate; manganese, 25 mg as manganous sulfate; iodine, 0.5 mg as calcium iodate; and selenium, 0.1 mg as sodium selenite.

**Table 2:** Analyzed chemical composition (g/kg) and gross energy (GE) values of co-products from wheat flour milling (ingredients and diets) DM basis.

	Shorts		Millrun							
Analyzed Content	A	B	A	B	C	D	E	Middlings	Bran	Corn
Ingredient										
Dry matter	901	899	904	891	892	904	902	886	910	854
Ash	73	55	75	65	54	62	52	53	67	21
Ether extract	29	34	56	48	45	41	44	41	30	33
Crude protein	278	249	200	187	171	190	165	221	159	94
Acid-detergent fiber	115	80	114	129	130	150	128	108	155	36
Neutral-detergent fiber	317	229	326	340	352	363	321	266	492	87
Crude fiber	79	52	77	83	88	99	82	71	120	18
GE (Mcal/kg DM)	4.56	4.57	4.55	4.55	4.57	4.56	4.56	4.60	4.53	4.35
Diet										
Dry matter	855	867	876	874	875	874	875	868	876	858
Ash	20	75	74	72	66	68	65	67	70	55
Ether extract	34	31	41	38	38	36	39	41	36	36
Crude protein	93	160	131	124	119	129	118	147	115	91
Acid-detergent fiber	34	66	71	75	81	82	73	66	79	42
Neutral-detergent fiber	87	165	187	202	197	196	195	164	240	98
Crude fiber	20	43	46	50	49	52	48	42	56	23
GE (Mcal/kg DM)	4.30	4.33	4.36	4.30	4.31	4.35	4.31	4.38	4.29	4.17

**Table 3:** In vitro digestibility and in vivo apparent total tract digestibility of gross energy (GE) and digestible energy value of co-products from wheat flour milling and corn (ingredients and diets) ^1^.

	Shorts		Millrun									
Characteristic	A	B	A	B	C	D	E	Middlings	Bran	Corn	SEM	*p*-Value
In vitro												
DM digestibility, %	65.2 ^b^	71.8 ^a^	60.7 ^d^	59.4 ^e^	58.7 ^ef^	58.3 ^f^	62.5 ^c^	71.1 ^a^	50.4 ^g^	NA	0.2	<0.001
GE digestibility, %	62.7 ^b^	69.1 ^a^	53.1 ^d^	51.6 ^e^	53.3 ^d^	53.5 ^d^	57.0 ^c^	68.0 ^a^	45.7 ^f^	NA	0.3	<0.001
In vivo ^2^												
Diet DE value, Mcal/kg DM	3.41 ^bcd^	3.56 ^a^	3.40 ^bcd^	3.39 ^cd^	3.31 ^de^	3.39 ^cd^	3.30 ^de^	3.53 ^ab^	3.21 ^e^	3.46 ^abc^	0.03	<0.001
ATTD of GE of ingredient, %	73.2 ^bc^	81.9 ^a^	70.5 ^c^	72.8 ^bc^	68.3 ^c^	70.5 ^c^	68.1 ^c^	77.7 ^ab^	62.6 ^d^	82.9 ^a^	1.1	<0.001
ATTD of GE of diet, %	78.8 ^bc^	82.5 ^a^	77.7 ^c^	78.7 ^bc^	76.8 ^c^	77.7 ^c^	76.7 ^c^	80.7 ^ab^	74.4 ^d^	82.9 ^a^	0.5	<0.001

^a–g^ Within a row, means without a common superscript differ (*p* < 0.05). ^1^ ATTD, apparent total tract digestibility; DE, digestible energy; DM, dry matter; NA, not available. ^2^ Means are based on 8 pig observation per diet.

**Table 4:** Characteristics of co-products (g/kg) from wheat flour milling and their relationship (Adj. R^2^) with in vitro and in vivo digestible energy (DE) values.

Variable	Mean	SD	CV	Lowest	Highest	Adj. R^2^ with In Vitro DE Value ^1^	Adj. R^2^ with In Vivo DE Value ^2^
Ash	62	9	14.4	52	75	0.02	0.06
Ether extract	41	9	21.2	29	56	0.07	0.14
Crude protein	202	40	19.8	160	278	0.53	0.48
Acid-detergent fiber	123	23	18.5	80	155	0.66	0.72
Neutral-detergent fiber	334	73	21.7	229	492	0.74	0.81
Crude fiber	83	19	22.4	52	120	0.66	0.78

^1^ Based on in vitro DE measurements of 94 co-products from wheat flour milling. ^2^ Based on in vivo DE measurements of 9 co-products from wheat flour milling.

**Table 5:** Kinetics and short-chain fatty acid (SCFA) production of in vitro fermentation of co-products from wheat flour milling ^1^.

	Shorts		Millrun					
Variable	A	B	B	D	Middlings	Bran	SEM ^2^	*p*-Value
Fermentation kinetics								
Lag time ^3^	4.01 ^ab^	3.95 ^b^	3.44 ^c^	3.45 ^c^	3.39 ^c^	4.29 ^a^	0.12	<0.001
Half time ^4^	11.5 ^a^	10.2 ^b^	10.2 ^b^	10.4 ^b^	9.82 ^b^	10.1 ^b^	1.89	<0.001
FRD ^5^	0.075 ^c^	0.097 ^ab^	0.085 ^bc^	0.093 ^b^	0.094 ^b^	0.111 ^a^	0.028	<0.001
Total gas ^6^	136 ^b^	147 ^a^	125 ^c^	101 ^d^	148 ^a^	125 ^c^	10.4	<0.001
Total SCFA ^7^	2.6 ^ab^	3.0 ^a^	2.1 ^b^	2.0 ^b^	2.9 ^a^	2.7 ^ab^	0.2	<0.001
Individual SCFA ^8^								
Acetate	60.8 ^a^	57.5 ^b^	61.4 ^a^	60.8 ^a^	60.8 ^a^	59.9 ^a^	1.1	<0.001
Propionate	24.6 ^c^	25.8 ^ab^	25.3 ^bc^	25.6 ^ab^	26.4 ^a^	25.3 ^bc^	1.6	<0.001
Butyrate	10.2 ^c^	12.7 ^a^	10.3 ^bc^	10.4 ^bc^	9.8 ^c^	10.9 ^b^	0.7	<0.001
BCFA	2.9 ^a^	2.7 ^ab^	2.1 ^bc^	2.0 ^c^	1.9 ^c^	2.7 ^ab^	0.1	<0.001

^a–d^ Within a row, means without a common superscript differ (*p* < 0.05). ^1^ BCFA, branched-chain fatty acid (sum of iso-butyric acid and iso-valeric acid); FRD, fractional rate of degradation; SCFA, short-chain fatty acid. ^2^ Number of observations in fermentation for each substrate = 12. ^3^ L, lag time, time taken to start fermentation (h). ^4^ T/2, half-time to asymptote (h). ^5^ *μ*, fractional rate of degradation (h^−1^) at *t* = *T*/2. ^6^ G_f_, cumulative maximum gas volume (ml per g sample incubated for fermentation). ^7^ mMol/g sample incubated for fermentation. ^8^ Molar ratio (%) of total SCFA.

## Data Availability

The original contributions presented in this study are included in the article. Further inquiries can be directed to the corresponding authors.
